# Clinical Application of Circulating Tumor Cells and Circulating Tumor DNA in Uveal Melanoma

**DOI:** 10.1200/PO.17.00279

**Published:** 2018-05-17

**Authors:** Aaron Beasley, Timothy Isaacs, Muhammad A. Khattak, James B. Freeman, Richard Allcock, Fred K. Chen, Michelle R. Pereira, Kyle Yau, Jaqueline Bentel, Tersia Vermeulen, Leslie Calapre, Michael Millward, Melanie R. Ziman, Elin S. Gray

**Affiliations:** **Aaron Beasley**, **Muhammad A. Khattak**, **James B. Freeman**, **Michelle R. Pereira**, **Leslie Calapre**, **Melanie R. Ziman**, and **Elin S. Gray**, Edith Cowan University, Joondalup; **Timothy Isaacs**, **Muhammad A. Khattak**, **Richard Allcock**, **Fred K. Chen**, **Kyle Yau**, **Michael Millward**, **Melanie R. Ziman**, and **Elin S. Gray**, University of Western Australia, Crawley; **Richard Allcock** and **Michael Millward**, Sir Charles Gairdner Hospital; **Fred K. Chen**, Lions Eye Institute, Nedlands; **Timothy Isaacs** and **Fred K. Chen**, Royal Perth Hospital, Perth; **Timothy Isaacs**, Perth Retina, West Leederville; and **Muhammad A. Khattak**, **Jaqueline Bentel**, and **Tersia Vermeulen** Fiona Stanley Hospital, Murdoch, Western Australia, Australia.

## Abstract

**Purpose:**

To evaluate the feasibility of using circulating tumor cells (CTCs) and circulating tumor DNA (ctDNA) for the management of uveal melanoma (UM).

**Patients and Methods:**

Low-coverage whole-genome sequencing was used to determine somatic chromosomal copy number alterations (SCNAs) in primary UM tumors, ctDNA, and whole-genome amplified CTCs. CTCs were immunocaptured using an antimelanoma-associated chondroitin sulfate antibody conjugated to magnetic beads and immunostained for melanoma antigen recognised by T cells 1 (MART1)/glycoprotein 100 (gp100)/S100 calcium-binding protein β (S100β). ctDNA was quantified using droplet digital polymerase chain reaction assay for mutations in the *GNAQ*, *GNA11*, *PLCβ4*, and *CYSLTR2* genes.

**Results:**

SCNA analysis of CTCs and ctDNA isolated from a patient with metastatic UM showed good concordance with the enucleated primary tumor. In a cohort of 30 patients with primary UM, CTCs were detected in 58% of patients (one to 37 CTCs per 8 mL of blood), whereas only 26% of patients had detectable ctDNA (1.6 to 29 copies/mL). The presence of CTCs or ctDNA was not associated with tumor size or other prognostic markers. However, the frequent detection of CTCs in patients with early-stage UM supports a model in which CTCs can be used to derive tumor-specific SCNA relevant for prognosis. Monitoring of ctDNA after treatment of the primary tumor allowed detection of metastatic disease earlier than ^18^F-labeled fluorodeoxyglucose positron emission tomography in two patients.

**Conclusion:**

The presence of CTCs in localized UM can be used to ascertain prognostic SCNA, whereas ctDNA can be used to monitor patients for early signs of metastatic disease. This study paves the way for the analysis of CTCs and ctDNA as a liquid biopsy that will assist with treatment decisions in patients with UM.

## INTRODUCTION

Uveal melanoma (UM) is the most common intraocular malignancy.^1^ Despite successful control of the primary tumor within the eye, metastatic disease ultimately develops in up to 50% of patients, predominantly in the liver. Currently, there are limited therapeutic options for metastatic UM, and as a result there is a high mortality rate.^2^ Extensive analysis of primary UMs has defined molecular features of the tumor cells that predict, with a high degree of accuracy, a patient’s risk for development of metastases. Biomarkers of poor prognosis include histopathological features of the tumor; somatic copy number alterations (SCNAs), such as loss of chromosome 3, 6q, and 8q^3,4^; *BAP1* mutations^5,6^; and the differential expression of marker genes that include well-characterized cancer-associated factors.^7,8^ Other features, such as gain in 6p and mutations in *EIF1AX* and *SF3B1*, are associated with better prognosis.^4,6^

Given that metastasis in UM arises from hematogenous dissemination, investigation of circulating tumor cells (CTCs) and circulating tumor DNA (ctDNA) could provide a unique opportunity for genetic analysis of the patient’s tumor through a simple and safe blood test. Previous research has indicated that CTCs harbor genetic profiles representative of the primary tumor^9^ and can be used to detect tumor-specific mutations in other cancers.^9-14^ In UM, amplification of DNA from a single CTC has been reported, with comparative genomic hybridization array showing copy number abnormalities associated with poor prognosis.^15^ A recent study described the detection of loss in chromosome 3 in CTCs using a modified fluorescence in situ hybridization technique as a method to aid prognostication of UM patients likely to metastasize.^16^

An additional blood-based marker, commonly used to evaluate tumor burden and tumor-specific genetic features, is ctDNA.^17,18^ The high proportion of recurrent hot spot mutations in UM enables the opportune detection of ctDNA using droplet digital polymerase chain reaction (PCR). UMs carry mutually exclusive activating mutations in *GNAQ*, *GNA11*, *PLCβ4*, and *CYSLTR2*, encompassing more than 90% of UM patients.^4,19-22^ Bidard et al^18^ detected ctDNA in 84% of patients with UM with metastatic disease and found ctDNA levels to be an independent prognostic factor for both progression-free survival and overall survival.^18^ However, the presence and prognostic significance of ctDNA in patients with primary UM without detectable metastatic disease has yet to be evaluated.

Given that UM harbors distinct SCNAs that correlate with poor prognosis (L1p, L3, L6q, G8q) and good prognosis (G6p),^4^ their detection in CTCs may offer a minimally invasive method for prognostication. Furthermore, because ctDNA is highly correlated with tumor volume,^18^ it may offer a minimally invasive method for detection of metastatic disease.

Here, we evaluated the viability of CTC and ctDNA as suitable biomarkers to derive prognostic information in UM. Whole-genome SCNAs were derived from CTCs and ctDNA from a patient with metastatic UM and compared with those in the primary tumor. The blood of patients with primary UM was analyzed for both the number of CTCs immunomagnetically captured using melanoma-associated chondroitin sulfate proteoglycan (MCSP) and the level of plasma ctDNA. Finally, we showed that ctDNA monitoring allowed early detection of metastatic disease in two patients with UM.

## PATIENTS AND METHODS

### Patients and Sample Processing

Thirty patients with primary UM from the Lions Eye Institute and Royal Perth Hospital, Perth, Western Australia, were enrolled in the study between March 2014 and November 2016. UM was diagnosed by clinical and ultrasound examination performed by a specialist ophthalmologist to evaluate the size and location of the intraocular tumor, including the presence of ciliary body involvement. Peripheral blood samples were taken from 30 patients before radiation plaque insertion or enucleation. Eight patients with metastatic UM were recruited from oncology outpatient clinics at Sir Charles Gardner and Fiona Stanley Hospitals. Written informed consent was obtained from all patients under approved Human Research Ethics Committee protocols from Edith Cowan University (No. 11543) and Sir Charles Gardner Hospital (No. 2013-246), Western Australia. For CTC quantification, blood was collected in Vacutainer K2 EDTA tubes (BD Biosciences, Franklin Lakes, NJ), stored at 4°C, and processed within 24 hours. Plasma was isolated by double centrifugation at 1,600 g for 10 minutes and stored at −80°C.

### 

### CTC Capture, Quantification, and Low-Pass Whole-Genome Sequencing

CTC isolation was adapted from a previously described protocol,^23^ detailed in the Data Supplement. One hundred nanograms of whole-genome amplified (WGA) DNA (Data Supplement) was used to construct 200-bp sequencing libraries using an NEB Next Ultra Fragment Library Kit (New England Biosciences, Ipswich, MA) and barcoded using the Ion Xpress Barcode Adapters 1-96 Kit (Life Technologies, Carlsbad, CA). Libraries were sequenced for 520 flows on a P1 sequencing chip using an Ion Proton Sequencer (Life Technologies). Sequencing depths ranged from 0.26 to 0.59×. Somatic mutations and SCNAs were analyzed using Ion Reporter 4.6 (Life Technologies).

### ctDNA Quantification

Cell-free DNA (cfDNA) was extracted from 1 to 5 mL of plasma using a QIAamp Circulating Nucleic Acid kit (Qiagen, Hilden, Germany) according to the manufacturer’s instructions and stored at −80°C. ctDNA was quantified using droplet digital PCR and PrimePCR assays as described in the Data Supplement.

### Statistical Analysis

The Spearman rank correlation coefficient was used to test the correlation between the level of ctDNA, number of CTCs, and tumor size. Tumor volume was calculated using a formula previously described.^24^ The numbers of captured CTCs in patients with and without monosomy of chromosome 3 were compared using a nonparametric Mann-Whitney *U* test. Statistical analyses were performed using Graphpad Prism version 5.0 (GraphPad Software, La Jolla, CA).

## RESULTS

### Analysis of Somatic Copy Number Alterations in CTCs From a Patient With UM With Metastatic Disease

**Fig 1.**
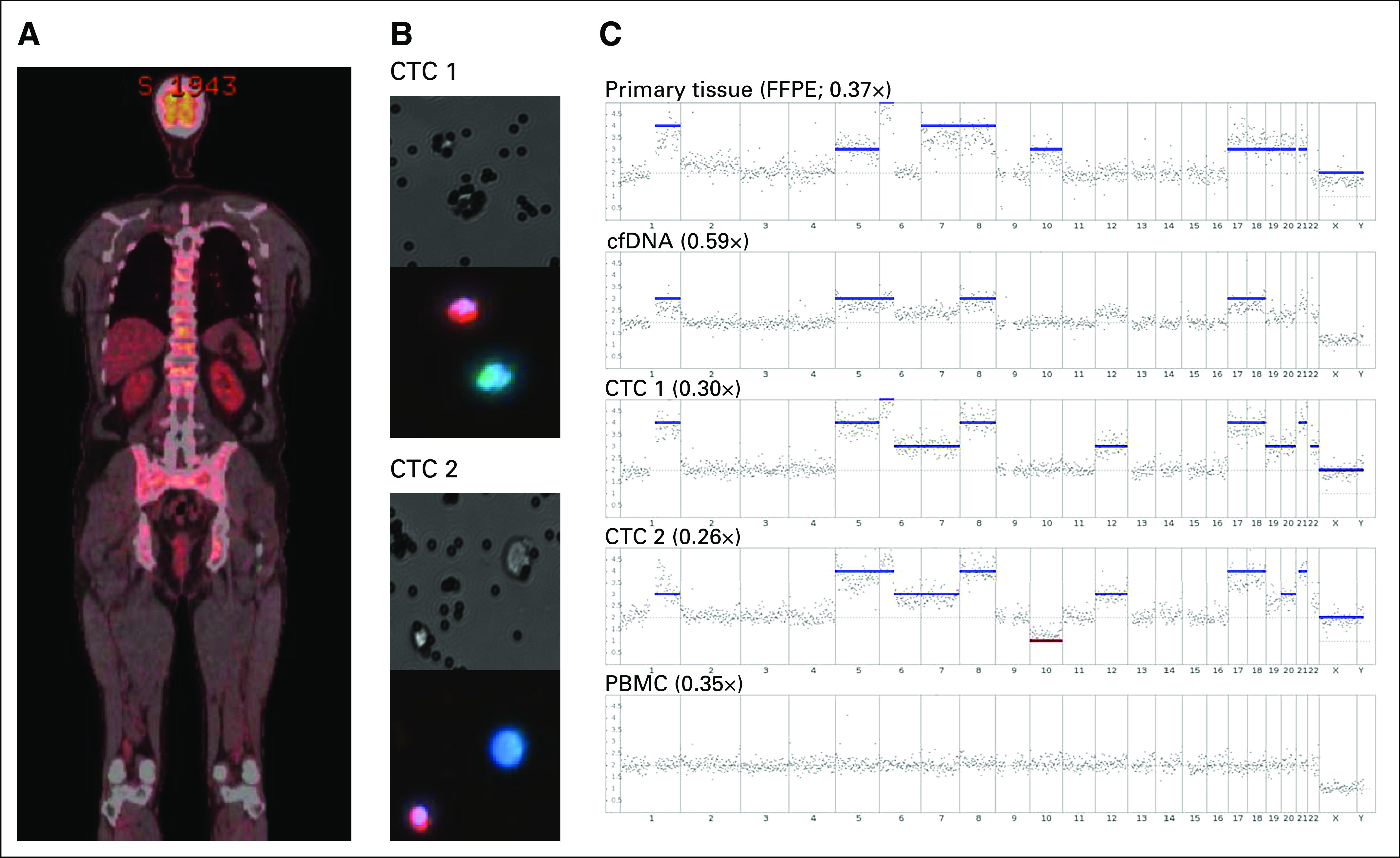
Comparison between the genetic profile of the primary tumor, cell-free DNA (cfDNA), two circulating tumor cells (CTCs), and a single peripheral blood mononuclear cell (PBMC) in a patient with metastatic uveal melanoma. (A) Whole-body fluorodeoxyglucose–positron emission tomography scan of a patient with uveal melanoma at the time of blood collection. (B) Brightfield and florescent images of the two CTCs used for somatic chromosomal copy number alteration analysis. Cells were stained with a combination of antibodies against the melanoma markers melanoma antigen recognised by T cells 1 (MART1)/glycoprotein 100(gp100)/S100 calcium-binding protein β (S100β; green), CD45 (red), and 4',6-diamidino-2-phenylindole (DAPI; blue), taken at ×200 magnification. (C) Whole-genome sequencing somatic chromosomal copy number alteration profile of primary formalin-fixed paraffin-embedded tumor, cfDNA, two CTCs, and a single PBMC. The obtained sequence depth is indicated for each plot. Red and blue bars represent chromosomal losses or gains, respectively.

CTCs were isolated from peripheral venous blood of a patient with metastatic UM (patient 640). The patient was originally diagnosed with UM in February 2015 and underwent plaque brachytherapy; however, response was suboptimal, and the eye was subsequently enucleated. The clinical history of this patient is detailed in the Data Supplement. In March 2017, the patient was diagnosed with metastatic UM, with bone marrow activity in the spine, pelvis, ribs, and both femora (Fig 1A). Analysis of a blood sample obtained in March 2017, before initiation of pembrolizumab therapy, showed the presence of 33 CTCs in 8 mL of blood. A total of three CTCs were separated and subjected to WGA (Fig 1B). A single peripheral blood mononuclear cell (PBMC) subjected to WGA was used as negative control. The accuracy and reliability of this WGA method for the analysis of single UM for assessment of SCNA were first demonstrated using individual cells from three UM cell lines (Data Supplement).

Two CTCs and a PBMC provided suitable WGA-DNA material for whole-genome sequencing. In addition, we sequenced DNA extracted from the patient’s archived formalin-fixed paraffin-embedded primary tumor (with > 80% tumor cellularity) taken 2 years before isolation of CTCs. We also assessed cfDNA extracted from the same blood draw as that for CTC isolation. The primary tumor had large copy number gains in chromosome 1q, 5, 6p, 7, 8, 10, 17, 18, 19, 20, 21, X, and Y (Fig 1C). Multiplex ligand-dependent probe amplification (MLPA) analysis of the primary tumor confirmed the chromosomal gains of 6p and 8p/8q and the lack of evidence for SCNAs of 1p and 3p/3q (Data Supplement). The two isolated CTCs also showed overlapping chromosomal gains and losses in comparison with the primary tumor, despite the primary tumor being removed 2 years earlier (Fig 1C). DNA was unable to be recovered from the bone metastases because of acid decalcification of the specimen before embedding. Additional alterations found in CTCs comprised a gain in 6q in both cells, a gain of chromosome 22 in CTC1, and a loss of chromosome 10 in CTC2. The PBMC analyzed did not harbor any SCNAs (Fig 1C), same as multiple PBMCs used as negative controls in the validation experiments (Data Supplement). The *GNA11* Q209L mutation was detectable in DNA derived from the primary tumor and in WGA CTCs.

Sequencing of cfDNA from the same blood sample from which CTCs were isolated also showed similar chromosome gains, with trends toward gains in some chromosomal alterations found in the primary tissue; however, these did not reach the threshold to be called a true gain (Fig 1C). cfDNA analysis could not detect all of the changes found in the primary tumor, most likely because of a high abundance of normal DNA. Droplet digital PCR targeting the *GNA11* Q209L mutation in the cfDNA indicated the presence of 15,460 copies/mL of mutant DNA in plasma, with a 20.2% frequency abundance relative to normal DNA. Thus, despite the significant abundance of ctDNA, this compartment is not as sensitive as CTCs for the analysis of SCNAs.

These results illustrate that tumor-associated SCNA can be ascertained through genetic analysis of CTCs, providing important prognostic information. Because prognostication is critical for the clinical management of patients diagnosed with early-stage UM, it was important to determine whether CTCs and ctDNA were readily detectable in these patients.

### CTCs in Blood of Patients With Primary UM

**Fig 2.**
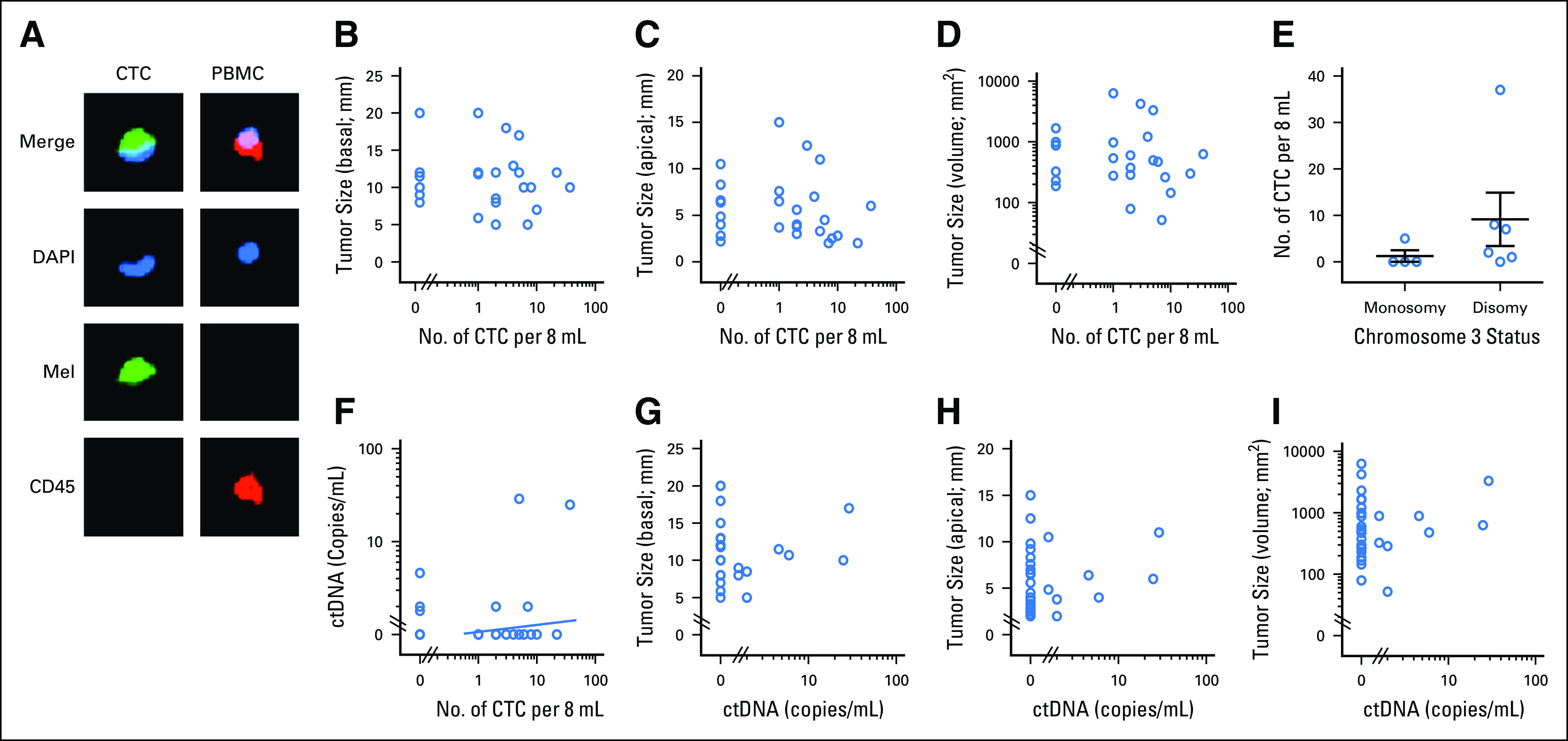
Circulating tumor cell (CTC) and circulating tumor DNA (ctDNA) quantification in a primary uveal melanoma (UM) cohort. (A) Example of immunocytochemical staining of a UM CTC and peripheral blood mononuclear cell (PBMC). Green fluorescence (AF488, Mel) indicates staining with a combination of antibodies against the melanoma markers MART1/gp100/S100β; red fluorescence (phycoerythrin [PE]) indicates CD45 positivity; and blue fluorescence (4',6-diamidino-2-phenylindole, DAPI) indicates the presence of a nucleus. CTCs were identified as Mel-positive and DAPI-positive and CD45-negative cells. (B-E) Graphs illustrate (B) CTC count versus basal median diameter (n = 26; *P* = .874; *r* = −0.034), (C) tumor size as apical height (n = 26; *P* = .237; *r* = −0.250), or (D) tumor volume (n = 26; *P* = .338; *r* = −0.244). Spearman rank *r* and *P* values are indicated. (E) Comparison of CTC counts in patients with UM with and without chromosome 3 monosomy (n = 10). Mann-Whitney *U* test *P* value is indicated. (F-I) Graphs illustrate (F) ctDNA copies/mL versus CTC count in 8 mL of blood (n = 26), (G) ctDNA copies/mL versus basal median diameter (n = 30; *P* = .787; *r* = 0.053), (H) tumor size as apical height (n = 30; *P* = .384; *r* = −0.170), or (I) tumor volume (n = 30; *P* = .982; *r* = −0.004). Spearman rank *r* and *P* values are indicated. No correlation was found between CTC, ctDNA, and tumor size.

To determine the UM CTC detection rate in patients with localized disease, we analyzed the blood from 30 patients with primary UM, without the presence of clinically identifiable metastatic disease, obtained before radiation plaque insertion or enucleation (Table 1). CTCs were identified by positive staining for melanoma antigen recognised by T cells 1 (MART1)/glycoprotein 100(gp100)/S100 calcium-binding protein β (S100β) and negative staining for CD45 (Fig 2A). A total of 15 of 26 (58%) individuals with assessable results had at least one CTC in 8 mL of blood, with a range of one to 37 CTCs detected, and 14 (54%) patients had two or more detectable CTCs. Only single cells, rather than clusters, were detected in all patients. The presence or quantity of CTCs captured using MCSP did not correlate with the tumor basal and apical sizes or tumor volume (Figs 2B-2D). Nevertheless, among the 10 patients who underwent tumor biopsy, no significant difference was found between the number of CTCs and monosomy or loss of chromosome 3 in the tumor (Fig 2E; *P* = .062).

**Table 1.**
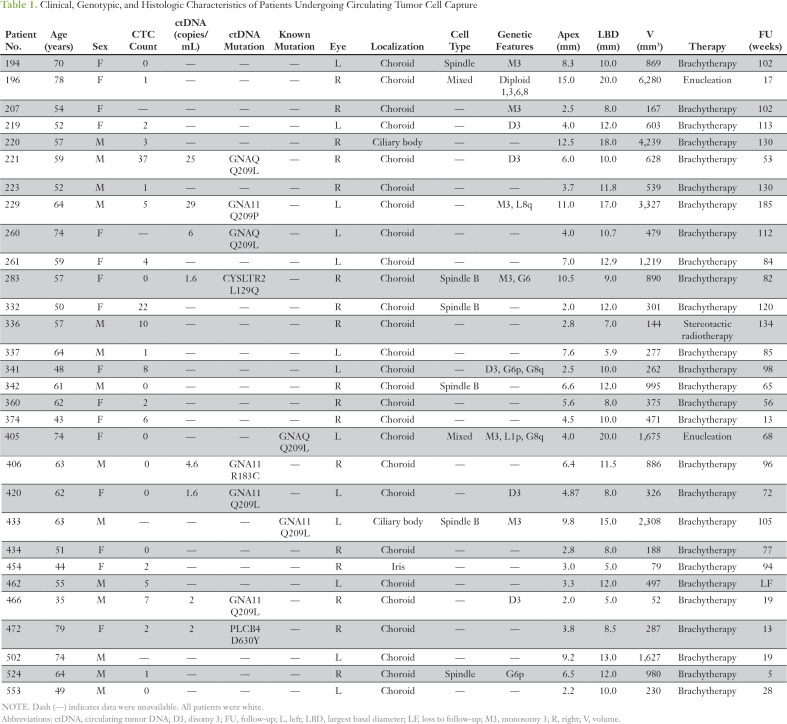
Clinical, Genotypic, and Histologic Characteristics of Patients Undergoing Circulating Tumor Cell Capture

### ctDNA in the Blood of Patients With Primary UM

The plasma from all 30 patients with UM described above was also analyzed for the presence of common UM-associated mutations *GNAQ*/*GNA11* Q209L/P, *GNAQ*/*GNA11* R183Q/C, *PLCβ4* D630Y, and *CYSLTR2* L129Q (Table 1; Fig 2). Screening for all of these mutations was necessary in our study, because for most patients fine-needle aspirate biopsies were either not performed or provided limited amounts of DNA that was used for MLPA testing and, therefore, we were unable to determine the tumor’s driver mutations before testing for ctDNA. Instead, all patient blood samples were tested for these mutations, because they have been reported to occur in > 90% of UMs.^19,20,22^ We detected ctDNA in eight of the 30 patients tested (23%; range, 1.6 to 29 copies); two patients had a *GNAQ* Q209L mutation, two had a *GNA11* Q209L mutation, one had a *GNA11* Q209P mutation, one had a *GNA11* R183C mutation, one had a *PLCβ4* D630Y mutation, and one had a *CYSLTR2* L129Q mutation. Only four of 30 patients had simultaneous detection of ctDNA and CTCs (Fig 2F). In those with detectable mutations, ctDNA levels were correlated with tumor size (largest basal/apical diameter/volume; Figs 2G-2I).

### ctDNA for Detection of Metastatic UM

**Fig 3.**
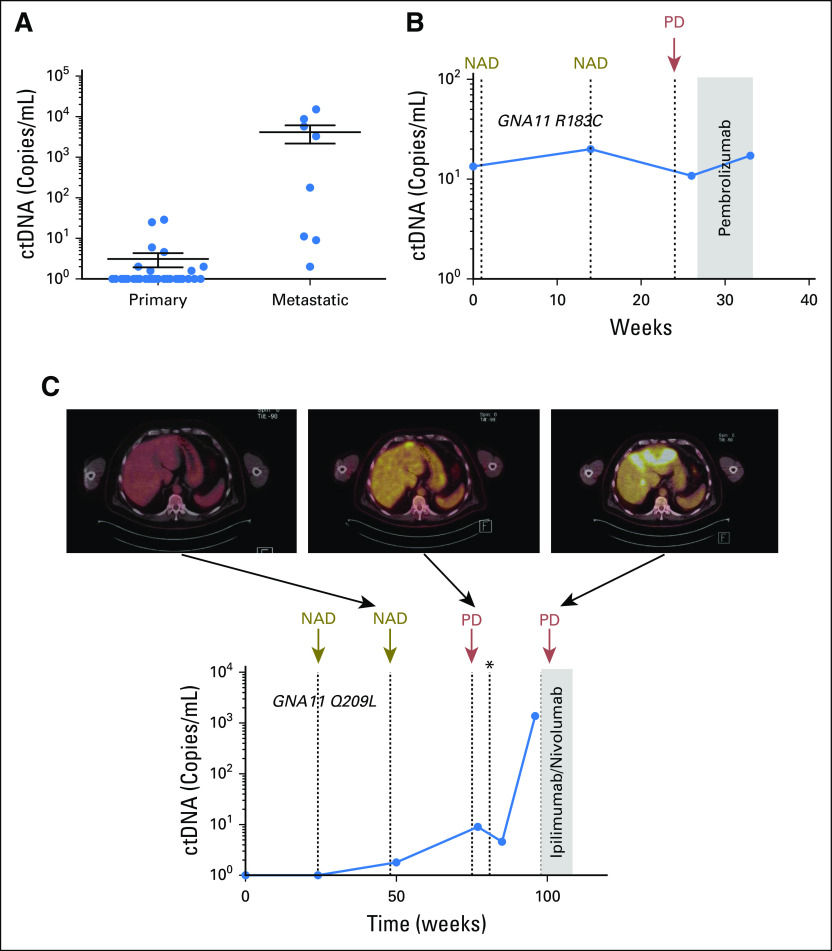
Analysis of circulating tumor DNA (ctDNA) in patients with liver metastasis. (A) Comparison of ctDNA levels in patients with primary (n = 27) and metastatic (n = 8) uveal melanoma (UM) showed statistically significant differences (Student *t* test, *P* < .001). (B) Plasma ctDNA levels in longitudinally collected samples from patient 656 with UM. (C) Plasma ctDNA levels in longitudinally collected samples from patient 433 with UM, before and after the development of overt metastatic disease as shown by fluorodeoxyglucose–positron emission tomography imaging of the liver. NAD, no active disease; PD, progressive disease. (*) Resection of a solitary liver metastasis.

We also analyzed ctDNA in eight patients with metastatic UM. In contrast to those with localized disease, all patients with metastatic UM had detectable ctDNA (Table 2; Fig 3A; *P* < .001). Retrospective analysis of longitudinal samples collected from two patients indicated that detection of ctDNA preceded radiologic recognition of liver metastases.

**Table 2.**
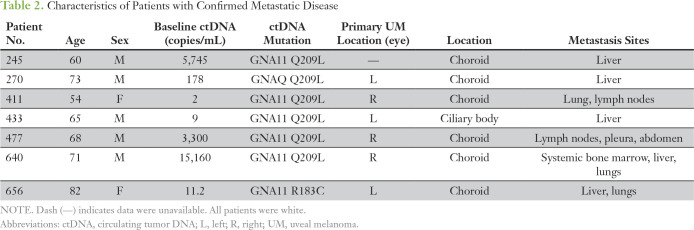
Characteristics of Patients with Confirmed Metastatic Disease

Patient 656 had an enucleation 3 months before enrollment and a history of low-grade lymphoproliferative disorder and pulmonary embolism. Pathology of the enucleated tumor confirmed a choroidal melanoma, with Callender classification mixed and no angiolymphatic invasion. MLPA classified this patient as high risk (loss of chromosome 3p/q plus gain of 8q). A positron emission tomography (PET) scan 4 weeks before enrollment showed mildly fluorodeoxyglucose (FDG)-avid bilateral pelvic and inguinofemoral lymphadenopathy consistent with her history of a low-grade lymphoproliferative disorder. Surveillance computed tomography scans performed 6 months later did not show any evidence of metastatic melanoma. However, the baseline liquid biopsy indicated the presence of mutant *GNA11* R183C ctDNA at 13 copies/mL (Fig 3B). A second blood test at week 13 showed a similar low level of ctDNA (20 copies/mL). A concurrent PET scan showed small-volume FDG-avid foci evident near the right hilum and at the right lower lung. An early follow-up computed tomography scan at week 24 confirmed multiple small moderate FDG-avid liver and lung metastases. The closest blood analysis, at week 26, before initiation of pembrolizumab therapy, still indicated low ctDNA concentrations (10.8 copies/mL). A final blood collection at week 33 indicated the presence of 17.2 copies of ctDNA, with a recent scan showing no improvement on disease burden.

Similarly, in another patient (patient 433), 1.8 copies/mL of ctDNA encoding a *GNA11* Q209L mutation was detected 55 weeks after brachytherapy (Fig 3C). A concurrent PET scan did not indicate the presence of metastatic growth. By week 75, a PET scan and MRI scan detected an isolated liver metastasis. Plasma analysis indicated an increase in ctDNA to 9 copies/mL. The metastatic lesion was surgically removed, and a slight decrease in ctDNA was observed. However, ctDNA levels significantly increased to 1,380 copies/mL by week 96, with the concurrent imaging indicating new extensive metastases to the left lobe of the liver and new bone metastases. These results illustrate that for UM, ctDNA can be used to track disease burden and has the potential to provide a complementary measure in addition to imaging.

## DISCUSSION

Our results provide the proof of concept for using blood-based biomarkers for prognostication and routine monitoring of patients with UM. First, we showed that SCNA significant for UM prognostication can be detected in CTCs. Second, we found that CTCs can be detected, albeit at a low quantity, in most patients with UM with primary localized disease. Finally, although ctDNA was commonly undetectable in localized UM, monitoring of ctDNA allowed for early detection of metastatic disease. These preliminary findings warrant additional clinical studies to validate the use of these two biomarkers for the management of UM.

Previously, SCNA profiles of the primary tumor have been shown to accurately identify patients with UM at risk for developing metastatic disease.^4,25^ Although fine-needle aspirate biopsies are performed worldwide, genetic testing of UM primary tumors can be hampered by patients declining biopsy because of the perceived invasiveness of the procedure and the preferred use of sight-conserving therapies. Nevertheless, up to 50% of patients with UM will develop metastatic tumors after either short or long latency periods.^26^ Thus, development of pre-emptive adjuvant therapies may be an important strategy for improving survival. In this context, the provision of a blood test for the identification of prognostic SCNA from CTCs would allow identification of patients at risk, aiding triaging of patients for clinical trials and more frequent systemic surveillance for metastatic disease.

Most CTC studies in UM have been limited to CTC quantification. However, they have failed to find significant association between the levels of CTCs and disease prognosis.^27,28^ Similarly, we found that most patients have detectable CTCs irrespective of the predicted propensity of their tumor to metastasize, indicating that the presence of these CTCs may not be associated with metastatic disease risk. However, our CTC detection was restricted to the detection of MCSP-expressing cells, and thus we cannot exclude that CTCs expressing other cell surface markers may also provide prognostic information, as shown in some studies.^28,29^ Nevertheless, the opportunity to examine the genomic features in CTCs may offer a more accurate indication of patient metastatic risk, in comparison with simple quantification of CTCs. On the basis of the results presented here, methodologies to enhance CTC capture^16,28^ are needed for the optimal implementation of CTCs as a viable alternate source of tumor genetic material from which prognosis can be derived for most patients.

Sequencing of the matching cfDNA revealed several large chromosomal gains, but because of the high abundance of normal cfDNA, we could only detect trends toward chromosomal amplifications found in the CTCs and primary tumor. Patient 640 (Fig 1) had a high disease volume, with 15,460 copies/mL of *GNA11* Q209L ctDNA with a fractional abundance of 20%, whereas by contrast the highest ctDNA level in our primary UM study was found to be 29 copies/mL, with a fractional abundance of < 1%; therefore, sequencing of patients with localized disease for SCNA profiles may prove ineffective. Previous studies investigating SCNA in cfDNA similarly required the presence of a large fraction of ctDNA present to obtain results similar to the tissue of origin.^30^ Thus, although ctDNA is much easier to isolate, analysis of SCNA in CTCs should prove to be a more effective means of analyzing prognostic SCNA.

We also showed that ctDNA is not commonly detectable in blood of patients with localized UM. In contrast, most patients with metastatic UM we have tested have detectable ctDNA, consistent with a previous report.^18^ Nonetheless, longitudinal monitoring of two patients with primary UM who later developed metastatic disease showed undetectable levels of ctDNA at baseline, but ctDNA was detected around the time of clinical confirmation of disease progression by PET scan. Our findings in this study indicate that the low levels of detectable ctDNA in patients with primary disease are not suitable for screening of patients at a high risk of developing metastasis. However, given the high proportion of hotspot mutations in UM,^4^ ctDNA analysis may be a feasible minimally invasive method of monitoring metastatic disease burden and disease progression, as we have exemplified here.

In conclusion, our study underscores the potential clinical use of liquid biopsy for UM. Possible clinical applications involve the use of CTCs to derive SCNA for stratification of UMs into low- or high-risk categories and ctDNA to monitor disease progression, pending clinical validation of our findings through future prospective studies.
